# Level of Agreement between the MotionMetrix System and an Optoelectronic Motion Capture System for Walking and Running Gait Measurements

**DOI:** 10.3390/s23104576

**Published:** 2023-05-09

**Authors:** Diego Jaén-Carrillo, Felipe García-Pinillos, José M. Chicano-Gutiérrez, Alejandro Pérez-Castilla, Víctor Soto-Hermoso, Alejandro Molina-Molina, Santiago A. Ruiz-Alias

**Affiliations:** 1 Department of Sport Science, Universität Innsbruck, 6020 Innsbruck, Austria; 2 Department of Physical Education and Sports, Faculty of Sport Sciences, University of Granada, 18016 Granada, Spain; 3 Sport and Health University Research Institute (iMUDS), University of Granada, 18007 Granada, Spain; 4 Department of Physical Education, Sports and Recreation, Universidad de La Frontera, Temuco 1145, Chile; 5 Department of Education, Faculty of Education Sciences, University of Almería, 04120 Almería, Spain; 6 SPORT Research Group (CTS-1024), CERNEP Research Center, University of Almería, 04120 Almería, Spain; 7 Department of Physiotherapy, Universidad San Jorge, 50830 Zaragoza, Spain

**Keywords:** analysis, biomechanics, gait, markerless, motion, reliability

## Abstract

Markerless motion capture systems (MCS) have been developed as an alternative solution to overcome the limitations of 3D MCS as they provide a more practical and efficient setup process given, among other factors, the lack of sensors attached to the body. However, this might affect the accuracy of the measures recorded. Thus, this study is aimed at evaluating the level of agreement between a markerless MSC (i.e., MotionMetrix) and an optoelectronic MCS (i.e., Qualisys). For such purpose, 24 healthy young adults were assessed for walking (at 5 km/h) and running (at 10 and 15 km/h) in a single session. The parameters obtained from MotionMetrix and Qualisys were tested in terms of level of agreement. When walking at 5 km/h, the MotionMetrix system significantly underestimated the stance and swing phases, as well as the load and pre-swing phases (*p* < 0.05) reporting also relatively low systematic bias (i.e., ≤ −0.03 s) and standard error of the estimate (SEE) (i.e., ≤0.02 s). The level of agreement between measurements was perfect (r > 0.9) for step length left and cadence and very large (r > 0.7) for step time left, gait cycle, and stride length. Regarding running at 10 km/h, bias and SEE analysis revealed significant differences for most of the variables except for stride time, rate and length, swing knee flexion for both legs, and thigh flexion left. The level of agreement between measurements was very large (r > 0.7) for stride time and rate, stride length, and vertical displacement. At 15 km/h, bias and SEE revealed significant differences for vertical displacement, landing knee flexion for both legs, stance knee flexion left, thigh flexion, and extension for both legs. The level of agreement between measurements in running at 15 km/h was almost perfect (r > 0.9) when comparing Qualisys and MotionMetrix parameters for stride time and rate, and stride length. The agreement between the two motion capture systems varied for different variables and speeds of locomotion, with some variables demonstrating high agreement while others showed poor agreement. Nonetheless, the findings presented here suggest that the MotionMetrix system is a promising option for sports practitioners and clinicians interested in measuring gait variables, particularly in the contexts examined in the study.

## 1. Introduction

There are various technologies for analyzing gait and related parameters, including 3D motion capture systems (MCS), image processing, and wearable sensors [1,2,3]. MCS use various methods such as optical, magnetic, or inertial sensors to measure the position and orientation of markers placed on the objects or individuals. In the field of human biomechanics, the tracking of three-dimensional (3D) motion is usually accomplished through the attachment of retro-reflective markers to participants, which are then monitored using infrared cameras. This technique can detect marker locations with high precision, down to sub-millimeter levels within a specific capture volume. It is widely regarded as the closest alternative to the highly precise fluoroscopy method, which is considered the gold standard in this field [1]. The resulting data is used to create a digital representation of movement and can be used in various fields such as biomechanics, sports science, animation, or in clinical settings [2]. However, commercially available systems for this analysis often have limitations that make their practicality challenging.

The use of marker-based MCS has become widespread in both the research and diagnosis fields, but its application in places like patient homes, sports fields, and public spaces is limited by the need for very specific recording settings (i.e., number, placement, and features of cameras, calibration process, reflected markers attached to the individual’s body, and data analysis). Motion capture aims to measure the movement of the skeletal system. However, due to factors such as clothing, skin, and soft tissue movement, the retro-reflective markers placed on key bony landmarks like the lateral malleolus may move in relation to the underlying bones [3]. Various approaches have been employed to reduce this error, but they cannot completely eliminate it [4]. Therefore, to minimize this error source, participants are usually instructed to wear tight or minimal clothing. Additionally, during long data collection periods or when participants sweat excessively (e.g., during a maximal aerobic capacity running test), the likelihood of markers moving or falling off increases, which can compromise data accuracy.

The practicality and clinical adoption of marker-based motion capture is hindered by the considerable amount of time it takes to securely fix markers to participants. Even with a skilled researcher, this process can take between 20 to 30 min, not including the time needed to prepare the markers before data collection. Additionally, the presence of the markers on the participant’s body or clothing may make them aware of their presence, possibly affecting their natural movement.

Markerless MCS share similarities with marker-based systems as they aim to accurately and reliably measure the 3D motion of human segments. Both systems aim for the representation of the skeletal system with a simplified biomechanical model. Nonetheless, Markerless systems do not require markers to be placed on the participant, instead using synchronized 2D video cameras to achieve a 3D reconstruction. Markerless MCS have been developed as an alternative solution to overcome the limitations of marker-based MCS [5,6,7,8,9]. These systems eliminate the need for markers, providing a time-efficient and user-friendly option for capturing human motion data. Unlike marker-based MCS, markerless systems rely on image-based tracking and machine-learning algorithms to estimate the movement of body joints and segments [5,6,7,8,9]. This provides a more natural experience for the subject being recorded, as well as a more practical and less time-consuming setting-up process for the practitioner. Recent technological advancements in computational speed and a growing commercial demand for markerless MCS have led to the development of several software packages that are available for purchase, such as Theia3D [10], DariMotion, and MotionMetrix. Additionally, some markerless software packages, such as OpenPose [9] and OpenCap, are available as open-source software [11].

One commercially available markerless system is the MotionMetrix software (Mo-tionMetrix AB, Lidingö, Sweden). The reliability of the system has been tested by evaluating the test-retest results of all variables during walking (at 5 km/h) and running (at 10 and 15 km/h), and the results suggest that the system may be useful in settings where more sophisticated systems (such as 3D MCS) are not accessible, taking into account the limitations of the software itself (i.e., the reliability of some parameters may vary depending on walking and running velocity) [12]. Additionally, the agreement between spatio-temporal parameters measured by MotionMetrix to high-speed videoanalysis and OptoGait measurements has been previously assessed [13]. Valid measures for step frequency and step length were reported, but the MotionMetrix system tended to overestimate contact time (CT) and underestimate flight time (FT) [13]. 

Despite its popularity among researchers and widespread use by clinicians and sports practitioners, there is still doubt about the accuracy and consistency of the MotionMetrix system compared to a recognized standard 3D MCS. Therefore, this study aims to evaluate the level of agreement between the MotionMetrix system and a 3D MCS (Qualisys AB, Göteborg, Sweden) for walking (at 5 km/h) and running (at 10 and 15 km/h) in healthy adults. 

## 2. Materials and Methods

### 2.1. Participants

Twenty-four recreationally active young adults (16 men, 8 women; age = 22.7 ± 2.6 years; body height = 1.72 ± 0.10 m; body mass = 69.1 ± 11.7 kg; weekly training = 6.9 ± 2.4 h/week) [14] were included in the study. They declared to participate voluntarily in the study and being familiar with running on a treadmill and free from injuries and health problems. Each participant signed an informed consent form after being informed of the study’s objectives and procedures, and it was made clear that they could leave at any time. The study was conducted in accordance with the Declaration of Helsinki (2013) and was approved by the local university’s Ethics Board (No. 2546/CEIH/2022). 

### 2.2. Procedures

Subjects were asked to avoid any strenuous physical activity for at least 48 h before the data collection and came to the laboratory only once. During the test, they wore their typical running clothes and shoes and went through a walking and running protocol on a treadmill (WOODWAY Pro XL, Woodway, Inc., Waukesha, WI, USA) [12]. Before data collection, subjects had a minimum 8 min accommodation period on the treadmill at a self-selected pace [15]. Immediately after, the subjects went through a protocol where they walked and ran for one minute at speeds of 5, 10, and 15 km/h [12]. Data were only collected during the last 30 s of each bout to ensure the subjects had adjusted to the speed.

### 2.3. Materials and Testing

The body height (m) and body mass (kg) of each subject were measured employing a stadiometer (SECA 222, SECA, Corp., Hamburg, Germany) and a bioimpedance scale (Inbody 230, Inbody, Seoul, Republic of Korea), respectively.

**Figure 1:**
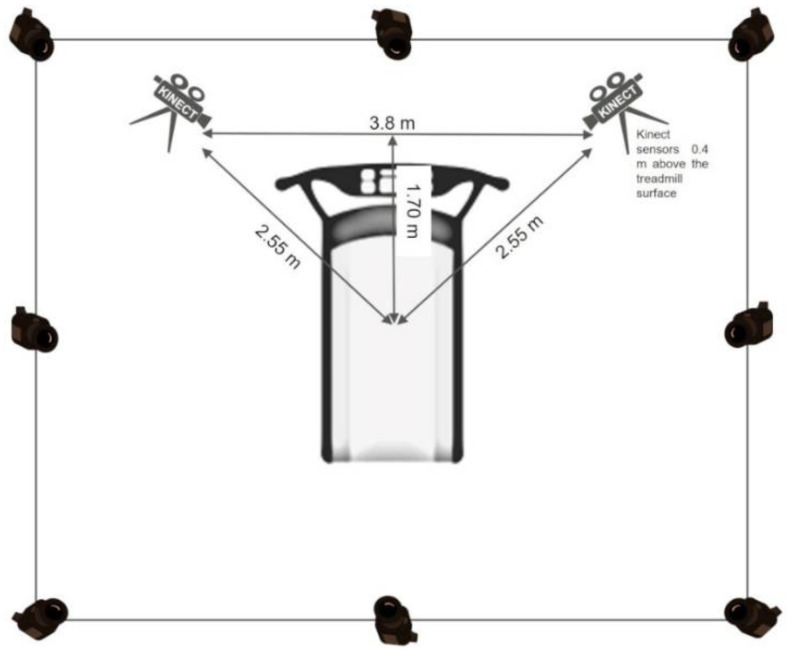
Diagram of the protocol setting for data acquisition displaying Kinect sensors and Qualisys cameras placement.

The MotionMetrix system was used in combination with two Kinect sensors (version 1.0, Microsoft, Washington, DC, USA) positioned on either side of the treadmill following a specific configuration (see Figure 1) as suggested by the manufacturer. The MotionMetrix system calculates various kinetic parameters based on the movement being analyzed (i.e., walking or running gait) [12]. The Kinect sensors, which have a depth sensor, allow for monitoring of 3D movements by recognizing 20 body joints in 3D space at a rate of 30 Hz. When both sensors track the same point simultaneously, this rate increases to 60 Hz. To ensure accurate data collection, the manufacturer’s guidelines were followed, including software calibration, wearing fitted clothing without shiny black fabric or reflective surfaces, securing shoelaces, tucking away hair, avoiding direct sunlight, and ensuring that treadmill parts do not obstruct the subject’s entire view. For a full description of how the MotionMetrix software provides measurements of the variables, the reader is pointed toward a previous study [12].

**Figure 2:**
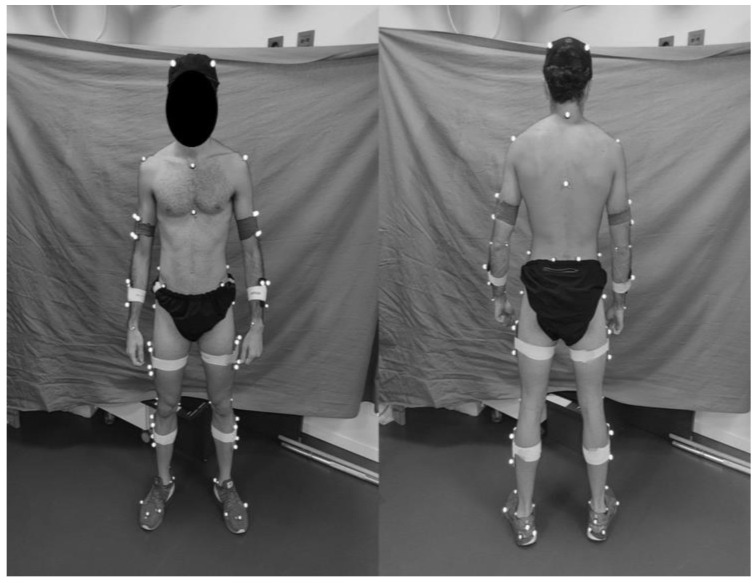
Marker set for data collection.

Eight Qualisys Oqus cameras (Qualisys AB, Gothenburg, Sweden) operating at 250 Hz and meticulously positioned to allow a full view of the treadmill (Figure 1) were employed for 3D motion capture analysis as the measure of reference. Before collecting data, the testing area was properly calibrated using a dynamic wand. After, 40 retro-reflective markers were attached to the subjects’ bodies to track 3D movement (Figure 2). Markers were placed on specific anatomical locations such as the right/left ilium crest tubercle, right/left posterior superior iliac spine, right/left femur greater trochanter, right/left anterior superior iliac spine, right/left femur lateral epicondyle, right/left femur medial epicondyle, right/left fibula apex of the lateral malleolus, right/left tibia apex of the medial malleolus, right/left head of the fifth metatarsals, right/left head of the first metatarsus, and right/left posterior surface of the calcaneus. Additionally, two marker sets were attached to the thigh and shank. After static calibration, the subjects performed the accommodation period to the treadmill and the entire protocol described above.

Visual 3D software (C-Motion Inc., Germantown, MD, USA) was used to process static and kinetic data. The motion data was processed using a low-pass filter with a cut-off frequency of 8 Hz to eliminate high-frequency noise. Joint angles were calculated using the x-y-z Cardan sequence, which represents flexion/extension, abduction/adduction, and axial rotation, without normalizing them to a static standing position. The laboratory frame was set up using the right-hand rule, with the positive y-direction facing forward, positive x-direction to the left, and positive z-direction upward. Variables of interest included the different phases of the gait cycle (walking at 5 km/h), stride rate and length, vertical displacement, spine angle, and landing, stance, and swing knee flexion (at 10 and 15 km/h).

### 2.4. Statistical Analysis

Descriptive data are presented as mean (±SD) and 95% confidence intervals (CI) The normal distribution of the data and equal distribution of variance were confirmed through the Shapiro–Wilk test and Levene’s test, respectively. A *t*-test pairwise mean comparison was performed to compare data obtained from MotionMetrix and Qualisys MCS. To assess the level of agreement between the two systems, Pearson’s product-moment correlation coefficient (r) was calculated between each variable. The correlation between measurements was interpreted using established criteria: <0.1 (trivial), 0.1–0.3 (small), 0.3–0.5 (moderate), 0.5–0.7 (large), 0.7–0.9 (very large), 0.9–1.0 (almost perfect) [16]. Additionally, intraclass correlation coefficients (ICC) were calculated. The authors followed the guidelines outlined by Koo and Li [17] and applied a “two-way random-effects” model (ICC [*2*,1]), using a “single measurement” type and “absolute agreement” definition for the ICC calculation. The benchmarks from Koo and Li [17] were used to interpret the ICC results: ICC values below 0.5 indicate ‘poor’ reliability, values between 0.5–0.75 represent ‘moderate’ reliability, 0.75–0.90 indicate ‘good’ reliability, and values above 0.90 signify ‘excellent’ reliability. The level of agreement between the MotionMetrix and Qualisys MCS was also evaluated through the systematic bias and the standard error of the estimate (SEE) from linear regression analysis. Statistical analysis was performed using the software package SPSS (IBM SPSS version 25.0, Chicago, IL, USA), and a significance level of *p* < 0.05 was established.

## 3. Results

### 3.1. Walking at 5 km/h

The pairwise comparison between the data obtained from the MotionMetrix and 3D MCS revealed significant differences for most of the variables, although the systematic bias and SEE were low (Table 1). 

Specifically, the MotionMetrix system significantly underestimated the stance and swing phases, as well as the load and pre-swing phases (*p* < 0.05). The systematic bias was relatively low (Bias ≤ −0.03 s) as well as the SEE (≤0.02 s).

However, the level of agreement between measurements when walking at 5 km/h (Table 1) was perfect (r > 0.9) for step length left and cadence. Very large (r > 0.7) when comparing Qualisys and MotionMetrix parameters for step time left, gait cycle, and stride length. Large agreements (r > 0.5) were revealed for step time right and step length right. Moreover, a moderate correlation (r > 0.4) was found for landing knee flexion for both legs. A small correlation (r > 0.1) for CT left, thigh extension left and thigh flexion right, and spine angle. Finally, a trivial correlation (r < 0.1) was found for CT right, step width, thigh flexion left, and thigh extension right. The ICCs also found a “good to excellent” association between MotionMetrix and Qualisys MCS measurements (ICCs > 0.75) for stride time, stride rate, stride length, and stance knee flexion left. Moderate agreements (ICCs > 0.642) were found for vertical displacement, stance knee flexion right, and swing knee flexion right. For the other variables, poor agreement (ICC < 0.5) was exhibited between both MCS.

### 3.2. Running at 10 km/h

Bias, obtained by pairwise comparison between data, and SEE for differences among MotionMetrix and Qualisys MCS revealed significant differences for most of the variables except for stride time, rate and length, swing knee flexion for both legs, and thigh flexion left (Table 2).

The level of agreement between measurements when running at 10 km/h (Table 2) was very large (r > 0.7) when comparing Qualisys and MotionMetrix parameters for stride time and rate, stride length, and vertical displacement. Large agreements (r > 0.5) were revealed for stance knee flexion and swing knee flexion for both legs. Moreover, a moderate correlation (r > 0.4) was found for landing knee flexion for both legs. A small correlation (r > 0.1) for CT left, thigh extension left and thigh flexion right, and spine angle. Finally, a trivial correlation (r < 0.1) was found for CT right, step width, thigh flexion left, and thigh extension right. The ICCs found a good association between MotionMetrix and Qualisys MCS measurements (ICCs > 0.75) for stride time, stride rate, stride length, and stance knee flexion left. Moderate agreements (ICCs > 0.642) were found for vertical displacement, stance knee flexion right, and swing knee flexion right. For the other variables, poor agreement (ICC < 0.5) was exhibited between both MCS.

### 3.3. Running at 15 km/h

Bias and SEE obtained by pairwise comparison between MotionMetrix and Qualisys MCS revealed significant differences for vertical displacement, landing knee flexion for both legs, stance knee flexion left, thigh flexion, and extension for both legs (Table 3).

The level of agreement between measurements in running at 15 km/h (Table 3) was almost perfect (r > 0.9) when comparing Qualisys and MotionMetrix parameters for stride time and rate and stride length. Very large agreements (r > 0.7) were revealed for vertical displacement and swing knee flexion for both legs. In addition, large correlations (r > 0.5) were found in the consistency between measurements for stance knee flexion in both legs. Moreover, a moderate correlation (r > 0.35) was found for CT for both legs and thigh flexion left. A small correlation (r > 0.1) for landing knee flexion for both legs, thigh extension left and thigh flexion right was revealed. Finally, a trivial correlation (r < 0.1) was found for step width, thigh extension right, and spine angle. The ICCs also found a “good to excellent” association between MotionMetrix and Qualisys MCS measurements (ICCs > 0.84) for Swing knee flexion for both legs, stride time, stride rate, and stride length. Moderate agreements were found for stance, knee flexion for both legs, and vertical displacement (ICCs > 0.617). For the other variables, poor agreement (ICCs < 0.5) was reflected between both MCS.

## 4. Discussion

This study is the first, to the best of the authors’ knowledge, to assess the level of agreement between the MotionMetrix system and a 3D MCS for walking and running kinetic parameters. The study involved 24 healthy young adults to achieve this aim. The MotionMetrix system demonstrated the highest level of agreement, indicating excellent agreement (ICC > 0.9), for the left step time and cadence, compared to Qualisys MCS, while walking at a speed of 5 km/h. Regarding the agreement of other variables, the text highlights that stride rate and length showed good agreement, with ICCs above 0.75, while gait cycle exhibited a moderate level of agreement, with ICCs exceeding 0.5. However, the other variables demonstrated poor agreement, with ICCs below 0.5. Concerning running at a speed of 10 km/h, both MCSs demonstrated good levels of agreement for stride time, stride rate, and knee flexion angles (stance and swing) on the left side. The agreement was also good for stride length. Conversely, the other variables had moderate to poor levels of agreement. At a faster running speed of 15 km/h, both MCSs demonstrated excellent levels of agreement for stride time, stride rate, stride length, and swing knee flexion on the left side. A good level of agreement was also observed for swing knee flexion on the right side, while moderate agreement was found for vertical displacement and knee flexion angles on the stance side. However, the ICCs for the other variables indicated poor levels of agreement.

Assessing walking and running gait can be important for a variety of reasons, including diagnosing and monitoring certain conditions and injuries, evaluating the effectiveness of interventions, and tracking changes over time. However, the accuracy and reliability of gait measurements depend on the selection of appropriate variables and the quality of the measurement instruments used.

Previous research has shown that the MotionMetrix system provides reliable measures for walking at 5 km/h of all parameters except for step width [12]. Our study supports these findings, as we found that step width measurements obtained with the MotionMetrix system showed poor reliability (ICC = 0.000) and significant differences (*p* < 0.05) when compared to the gold standard system (i.e., Qualisys MCS) during walking at 5 km/h. It is important to note that the MotionMetrix system utilized two Kinect cameras and software to evaluate walking and running gait in this study. Prior research has demonstrated that the Kinect cameras have underestimated step time and step length by 16% and 1.7%, respectively, during walking [18]. Our findings partially refute this statement since no significant differences were observed for step time (*p* > 0.05, Bias = 0.00 s). Although significant differences were detected for step length when measured unilaterally, no significant differences were observed when measured as stride length (*p* > 0.05, Bias = 0.00 m), that is, one step after the other.

Additionally, the aforementioned study investigated the test-retest reliability of MotionMetrix for analyzing running at 10 and 15 km/h [12]. These authors reported reliable measures for all its parameters at 10 km/h, except for thigh flexion, landing knee flexion, and step width, which exhibited coefficients of variation (CV) of 16.26%, 10.14%, and 10.72%, respectively [12]. Consistent with the earlier investigation, the current findings showed significant differences (*p* < 0.05) between MotionMetrix and Qualisys for thigh flexion in both legs (Bias = 5.04° and 6.38°, left and right legs, respectively), landing knee flexion (Bias = −4.83° and 3.17°, left and right legs, respectively), and step width (Bias = 0.03 m) at 10 km/h. At 15 km/h, the previous study found that MotionMetrix provided reliable values for all parameters except spine angle and step width, which exhibited CVs of 23.27% and 22.22%, respectively [12]. In the current investigation, large SEE values were found between MotionMetrix and 3D MCS for spine angle (SEE = 3.40) and step width (SEE = 0.02).

When interpreting the findings here reported, readers must be aware of certain limitations. It is important to note that the treadmill protocol employed in this study aimed to minimize gait and running variability due to inexperience or fatigue [19,20]. Previous studies have shown that novice treadmill runners and healthy young adults require a minimum of 6 to 8 min to adjust to the treadmill’s locomotion. Running on a treadmill may not be the same as running on an overground outdoor surface, but they are largely comparable [15]. The present study was limited by its sample of healthy, active young adults, and as such, caution should be taken when generalizing the findings to other populations.

Despite the limitations exposed above, the study examined several variables that are commonly used to analyze gait patterns in walking and running, including step time, cadence, stride rate, stride length, and knee flexion angles. The study’s findings indicate that the MotionMetrix system is a reliable system for measuring these variables in both walking and running gait. These variables offer valuable information about the temporal and spatial aspects of gait, such as the duration of different phases, the frequency and distance of steps, and the angles of the joints involved in the movement. Accurate and reliable measurement of these variables can provide insights into the mechanics and efficiency of gait and help identify abnormalities or deviations from normal patterns. This information can guide the development of interventions, such as exercise programs or orthotics, to address gait impairments and improve functional outcomes. Therefore, considering these variables when assessing walking gait and running can provide valuable information for both research and clinical purposes, leading to a better understanding, diagnosis, and treatment of gait-related conditions and injuries.

## 5. Conclusions

After comparing the measurements of gait and running variables provided by the MotionMetrix markerless software against a gold standard system (i.e., Qualisys 3D MCS), it is concluded that the agreement between the two motion capture systems varied for different variables and speeds of locomotion, with some variables demonstrating high agreement while others showed poor agreement. Nonetheless, the findings presented here suggest that the MotionMetrix system is a promising option for sports practitioners and clinicians interested in measuring gait variables, particularly in the contexts examined in the study. However, further research is needed to examine the agreement between different systems for other variables and in different contexts.

## Figures and Tables

**Table 1:** Descriptive data (mean ± SD), bias, Pearson’s product-moment correlation coefficients (r), standard error of the estimate (SEE), and intraclass correlation coefficients (ICC [*2*,1]) for comparison between values obtained from MotionMetrix and Qualisys motion capture systems walking at 5 km/h.

Variable	Qualisys	MotionMetrix	Bias (95% CI) ^	r	SEE	ICC (95% CI)
Stance phase left (s)	0.68 (0.04)	0.65 (0.01)	−0.03 (−0.05 to −0.01) *	0.205	0.01	0.101 (−0.479 to 0.542)
Stance phase right (s)	0.68 (0.03)	0.65 (0.01)	−0.03 (−0.05 to −0.02) *	−0.029	0.04	−0.013 (−0.524 to 0.439)
Swing phase left (s)	0.36 (0.02)	0.35 (0.01)	−0.01 (−0.02 to 0.00) *	0.372	0.01	0.334 (−0.271 to 0.690)
Swing phase right (s)	0.36 (0.02)	0.35 (0.01)	−0.01 (−0.02 to −0.01) *	0.337	0.02	0.257 (−0.298 to 0.634)
Load response left (s)	0.16 (0.02)	0.15 (0.01)	−0.00 (−0.01 to 0.00)	0.365	0.02	0.446 (−0.290 to 0.767)
Load response right (s)	0.16 (0.01)	0.15 (0.02)	−0.01 (−0.02 to −0.00) *	0.180	0.01	0.173 (−0.347 to 0.576)
Pre-swing left (s)	0.16 (0.02)	0.15 (0.01)	−0.01 (−0.02 to 0.00) *	−0.005	0.02	−0.04 (−1.09 to 0.527)
Pre-swing right (s)	0.16 (0.01)	0.15 (0.01)	−0.01 (−0.02 to −0.00) *	0.149	0.02	0.186 (−0.581 to 0.629)
Total double support (s)	0.32 (0.03)	0.30 (0.01)	−0.02 (−0.03 to −0.00) *	0.236	0.03	0.247 (−0.440 to 0.654)
Step time left (s)	0.52 (0.02)	0.52 (0.02)	0.00 (−0.00 to 0.00)	0.920 **	0.01	0.960 (0.901 to 0.984)
Step time right (s)	0.52 (0.03)	0.52 (0.02)	0.00 (−0.01 to 0.01)	0.697 **	0.02	0.808 (0.531 to 0.922)
Gait cycle (s)	1.05 (0.04)	1.07 (0.12)	0.02 (−0.02 to 0.06)	0.785 **	0.03	0.686 (0.236 to 0.872)
Step length left (m)	0.87 (0.04)	0.73 (0.03)	−0.15 (−0.16 to −0.13) *	0.746 **	0.03	0.176 (−0.04 to 0.548)
Step length right (m)	0.57 (0.04)	0.72 (0.03)	0.15 (0.13 to 0.17) *	0.510 *	0.04	0.101 (−0.053 to 0.390)
Stride length (m)	1.44 (0.08)	1.46 (0.06)	0.01 (−0.01 to 0.03)	0.713 **	0.06	0.817 (0.563 to 0.924)
Cadence (spm)	114.5 (4.84)	114.4 (4.85)	−0.06 (−0.30 to 0.17)	0.994 **	0.56	0.997 (0.993 to 0.999)
Step width (m)	0.01 (0.00)	0.16 (0.04)	0.15 (0.13 to 0.16) *	−0.006	0.00	0.00 (−0.04 to 0.09)

%GT: percentage of gait cycle; spm: number steps per minute. ^ calculated by pairwise mean comparison (*t*-test). * *p* < 0.05. ** *p* < 0.001.

**Table 2:** Descriptive data (mean ± SD), bias, Pearson’s product-moment correlation coefficients (r), standard error of the estimate (SEE), and intraclass correlation coefficients (ICC [*2*,1]) for comparison between values obtained from MotionMetrix and Qualisys running at 10 km/h.

Variable	Qualisys	MotionMetrix	Bias (95% CI) ^	r	SEE	ICC (95% CI)
Stride time (s)	0.73 (0.04)	0.73 (0.05)	0.00 (−0.01 to 0.02)	0.770 **	0.03	0.873 (0.694 to 0.948)
Stride rate (spm)	82.22 (4.88)	82.13 (5.28)	−0.09 (−1.72 to 1.54)	0.741 **	3.64	0.855 (0.648 to 0.940)
Stride length (m)	2.03 (0.12)	2.04 (0.13)	0.00 (−0.04 to 0.04)	0.766 **	0.08	0.872 (0.688 to 0.947)
Contact time left (s)	0.25 (0.03)	0.30(0.03)	0.05 (0.03 to 0.07) *	0.107	0.03	0.096 (−0.285 to 0.473)
Contact time right (s)	0.25 (0.04)	0.28 (0.02)	0.03 (0.01 to 0.04) *	0.010	0.02	0.013 (−0.286 to 0.359)
Step width (m)	0.03 (0.01)	0.05 (0.02)	0.03 (0.01 to 0.04) *	−0.384	0.02	−0.419 (−1.169 to 0.335)
Vertical displacement (m)	0.10 (0.01)	0.08 (0.02)	−0.02 (−0.02 to −0.01) *	0.741 **	0.01	0.642 (−0.214 to 0.882)
Landing knee flexion left (°)	18.22 (6.84)	13.39 (2.94)	−4.83 (−7.53 to −2.12) *	0.452 *	2.69	0.382 (−0.225 to 0.719)
Landing knee flexion right (°)	15.24 (6.66)	18.42 (3.34)	3.17 (0.53 to 5.82) *	0.448 *	3.06	0.475 (−0.130 to 0.771)
Stance knee flexion left (°)	41.06 (5.60)	39.09 (4.11)	−1.97 (−3.8 to −0.13) *	0.673 **	3.11	0.752 (0.401 to 0.897)
Stance knee flexion right (°)	40.23 (4.87)	44.43 (4.71)	4.20 (2.49 to 5.91) *	0.676 **	3.55	0.661 (−0.132 to 0.883)
Swing knee flexion left (°)	90.46 (10.72)	89.87 (13.50)	−0.59 (−5.16 to 3.98)	0.622 *	10.18	0.803 (0.508 to 0.920)
Swing knee flexion right (°)	90.74 (9.80)	91.83 (15.90)	1.09 (−4.97 to 7.15)	0.519 *	13.93	0.643 (0.124 to 0.853)
Thigh flexion left (°)	18.94 (3.44)	23.98 (7.99)	5.04 (1.21 to 8.86)	0.023	8.18	0.026 (−0.782 to 0.534)
Thigh extension left (°)	−12.19 (14.00)	−26.48 (3.68)	−14.29 (−20.26 to −8.31) *	0.268	3.63	0.128 (−0.297 to 0.512)
Thigh flexion right (°)	17.93 (3.90)	24.31 (7.70)	6.38 (2.73 to 10.03) *	0.112	7.84	0.113 (−0.468 to 0.546)
Thigh extension right (°)	−17.28 (3.55)	−25.97 (4.20)	−8.68 (−11.04 to −6.33) *	0.070	4.30	0.039 (−0.169 to 0.328)
Spine angle (°)	4.11 (2.11)	6.99 (2.12)	2.88 (1.66 to 4.09) *	0.165	2.15	0.161 (−0.286 to 0.544)

Stpm: number of strides per minute; ^ calculated by pairwise mean comparison (*t*-test). * *p* < 0.05. ** *p* < 0.001.

**Table 3:** Descriptive data (mean ± SD), bias, Pearson’s product-moment correlation coefficients (r), standard error of the estimate (SEE), and intraclass correlation coefficients (ICC [*2*,1]) for comparison between values obtained from MotionMetrix and Qualisys running at 15 km/h.

Variable	Qualisys	MotionMetrix	Bias (95% CI) ^	r	SEE	ICC (95% CI)
Stride time (s)	0.67 (0.05)	0.67 (0.05)	0.00 (−0.00 to 0.00)	0.994 **	0.00	0.997 (0.993 to 0.999)
Stride rate (spm)	90.05 (6.50)	89.93 (6.50)	−0.11 (−0.40 to 0.17)	0.995 **	0.65	0.998 (0.994 to 0.999)
Stride length (m)	2.79 (0.21)	2.79 (0.20)	0.00 (−0.01 to 0.01)	0.994 **	0.02	0.997 (0.993 to 0.999)
Contact time left (s)	0.21 (0.02)	0.23 (0.01)	0.02 (0.01 to 0.03) *	0.387	0.01	0.313 (−0.251 to 0.673)
Contact time right (s)	0.21 (0.02)	0.22 (0.01)	0.02 (0.01 to 0.02) *	0.373	0.01	0.337 (−0.264 to 0.691)
Step width (m)	0.03 (0.02)	0.05 (0.03)	0.01 (0.00 to 0.03)	−0.533 *	0.02	−1.580 (−5.508 to −0.031)
Vertical displacement (m)	0.09 (0.01)	0.07 (0.02)	−0.02 (−0.01 to 0.03) *	0.747 **	0.01	0.617 (−0.238 to 0.877)
Landing knee flexion left (°)	18.41 (8.40)	14.31 (3.59)	−4.10 (−7.75 to −0.44) *	0.256	3.56	0.274 (−0.478 to 0.674)
Landing knee flexion right (°)	17.17 (8.40)	18.05 (2.91)	0.87 (−2.83 to 4.57) *	0.192	2.93	0.218 (−0.957 to 0.680)
Stance knee flexion left (°)	42.40 (5.37)	37.97 (4.73)	−4.42 (−6.34 to −2.50) *	0.639 **	3.73	0.634 (−0.116 to 0.868)
Stance knee flexion right (°)	41.51 (4.49)	42.93 (4.66)	1.42 (−0.43 to 3.27)	0.586 *	3.87	0.725 (0.359 to 0.884)
Swing knee flexion left (°)	112.93 (13.0)	112.42 (12.81)	−0.51 (−3.72 to 2.71)	0.842 **	3.87	0.918 (0.801 to 0.966)
Swing knee flexion right (°)	112.91 (13.08)	111.67 (10.59)	−1.24 (−5.18 to 2.70)	0.737 **	7.33	0.842 (0.620 to 0.934)
Thigh flexion left (°)	21.25 (10.92)	32.38 (5.88)	11.03 (6.41 to 15.64) *	0.354	5.63	0.288 (−0.250 to 0.653)
Thigh extension left (°)	−21.62 (10.35)	−34.57 (3.53)	−12.95 (−17.64 to −8.25) *	0.102	3.60	0.052 (−0.272 to 0.412)
Thigh flexion right (°)	22.66 (3.85)	34.03 (6.00)	11.37 (8.56 to 14.19) *	0.229	5.99	0.112 (−0.149 to 0.430)
Thigh extension right (°)	−20.75 (10.72)	−35.45 (4.49)	−14.69 (−20.09 to −9.29) *	−0.134	4.56	−0.078 (−0.424 to 0.322)
Spine angle (°)	4.69 (2.56)	6.72 (3.45)	2.03 (−0.11 to 4.17)	−0.277	3.40	−0.588 (−2.364 to 0.303)

Stpm: number of strides per minute; ^ calculated by pairwise mean comparison (*t*-test). * *p* < 0.05. ** *p* < 0.001.

## Data Availability

The data presented in this study are available upon request from the corresponding author. The data are not publicly available due to participant privacy.
